# Investigating the causal relationships between attention-deficit/hyperactivity disorder and autoimmune diseases: Evidence from Mendelian randomization study

**DOI:** 10.1097/MD.0000000000041157

**Published:** 2025-01-03

**Authors:** Yidong Zhou, Bowen Jin, Kai Qiao

**Affiliations:** a Department of Clinical Medicine, Fourth Clinical Medical College of Zhejiang Chinese Medical University, Hangzhou, Zhejiang, China; b Department of Medical Imaging Science, First Clinical Medical College of Zhejiang Chinese Medical University, Hangzhou Zhejiang, China.

**Keywords:** ADHD, autoimmune diseases, causal relationships, Mendelian randomization, psoriasis

## Abstract

Attention-deficit/hyperactivity disorder (ADHD) and autoimmune diseases have been found to be correlated in the observational studies, but the causal relationships have not been fully investigated. Two-sample Mendelian randomization (MR) analysis was used to explore the causal relationships between ADHD and 8 autoimmune disorders (systemic lupus erythematosus, Crohn disease, ulcerative colitis, type 1 diabetes, rheumatoid arthritis, psoriasis, ankylosing spondylitis [AS], and multiple sclerosis) with the publicly available genome-wide association study data in the European populations. Inverse-variance weighted (IVW), weighted median, and MR-Egger were used to estimate the causal effects. Extensive sensitivity analyses were employed to validate the 3 assumptions of MR and robustness of the results. Multivariable MR (MVMR) analysis was used to evaluate the direct causal effects adjusting for the potential confounding factors. The potential mediators of the causal effects were explored through the 2-step MR mediation analysis. With the Bonferroni corrected threshold, the IVW results indicated that genetically determined higher risk of ADHD was significantly associated with increased risk of psoriasis (IVW OR: 1.29; 95% CI: 1.11–1.49, *P* = 6.3e−04), but not with other autoimmune disorders. The reverse MR didn’t find significant causal effects of autoimmune diseases on ADHD. MVMR analysis indicated that the significant causal effects of ADHD on psoriasis remained significant after accounting for obesity, alcohol drinking, depression, and biological sex, but became nonsignificant when adjusting for smoking. Further mediation analysis suggested smoking might partially mediate the causal effects of ADHD on psoriasis (mediated percentage: 11.16%, 95% CI: 1.54% to 20.77%, *P* = .023). There is a significant causal relationship between ADHD and psoriasis, but not with other autoimmune disorders. The causal effects might be mediate by smoking. Our findings suggested that early prevention and lifestyle changes (such as smoking cessation) might be helpful to reduce the risk of developing psoriasis for ADHD patients. Further investigations were warranted to explore the underlying mechanisms and the potential clinical applications.

## 1. Introduction

Attention-deficit/hyperactivity disorder (ADHD) is a neurodevelopmental disorder with symptoms of inattention and/or hyperactivity-impulsivity in childhood may extend to adulthood in around 65% of the children diagnosed with ADHD.^[1]^ Individuals with ADHD may experience multiple social and emotional difficulties that seriously impact the normal life.^[2]^ ADHD is a highly heritable disorder, and the interactions between genetic and environmental risk factors might contribute to the pathogenesis of the disease.^[3]^

ADHD has been found to be associated with multiple psychiatric and nonmental disorders across the lifespan.^[4–7]^ As the role of immune alternations was being increasingly recognized in the development and progress of ADHD, growing studies have focused on the associations between ADHD and immune-mediated diseases especially autoimmune disorders in recent decades.^[8,9]^ An observational study of 983,680 Danish individuals found increased risk of ADHD in individuals with a personal or maternal history of autoimmune diseases such as type 1 diabetes, psoriasis, ankylosing spondylitis (AS).^[10]^ In another cross-sectional study of a cohort of 2,500,118 Norwegian individuals, significant associations between ADHD and common autoimmune conditions including psoriasis, Crohn disease (CD), and ulcerative colitis (UC) were reported, and the relation seems to be stronger in females.^[11]^ Although these observational studies have tried to control the available confounding factors, the residual confounding effects and reverse causation might still bias the associations,^[12]^ leaving the causal relationships between ADHD and autoimmune diseases that remained unclear.

Mendelian randomization (MR) was designed to explore the causal relationships between exposure and outcome variables through genetic instruments.^[13]^ The confounding effects and reverse causation bias of observational studies were minimized in the MR analysis since the genetic variants were determined and randomly distributed to the offspring at conception.^[14]^ MR has been widely used to infer the causal effects of ADHD on multiple outcomes such as other mental disorders,^[15]^ neurocognitive features,^[16]^ substance use,^[17]^ and physical health.^[18]^ However, the causal relationships between ADHD and autoimmune diseases have not been fully investigated.

**Figure 1.**
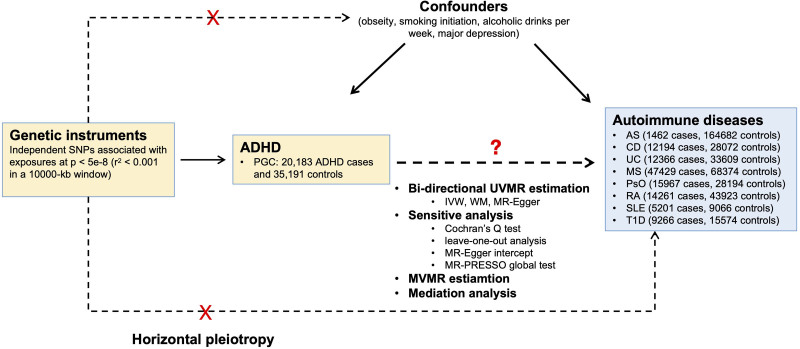
Overview of the study. PGC = psychiatric genomics consortium, SLE = systemic lupus erythematosus, CD = Crohn disease, UC = ulcerative colitis, T1D = type 1 diabetes, RA = rheumatoid arthritis, PsO = psoriasis, AS = ankylosing spondylitis, MS = multiple sclerosis.

The objective of this study is to investigate the causal relationships between ADHD and 8 common autoimmune diseases including AS, CD, UC, multiple sclerosis (MS), psoriasis (PsO), rheumatoid arthritis, systemic lupus erythematosus (SLE), and type 1 diabetes (T1D) by 2-sample MR analysis. For the significant causal effects identified, further multivariable MR and mediation MR analyses were conducted to explore the role of potential mediators in the causal effects. The overview of the study design was shown in Figure 1.

## 2. Materials and methods

To investigate the causal relationships between ADHD and the autoimmune diseases, the 2-sample MR study was conducted with 3 key assumptions^[19]^: the genetic variants are strongly correlated with the exposure variables (relevance assumption); there are no confounding factors of the associations between genetic variants and the outcome variables (independence assumption); the genetic variants only affect the outcome through exposure (exclusion-restriction assumption).

### 2.1. Data sources

Summary level genome-wide association study (GWAS) data for ADHD were obtained from a meta-analysis of 12 cohorts consisting of 55,374 participants (20,183 ADHD cases and 35,191 controls) by the psychiatric genomics consortium.^[20]^ Individuals with ADHD were identified through psychiatric interview or diagnosis by qualified psychiatrists. Genetic principle components and cohort specific covariates were adjusted for the GWAS analysis.

Summary GWAS data for the autoimmune diseases including AS,^[21]^ Crohn disease,^[22]^ UC,^[22]^ MS,^[23]^ psoriasis,^[24]^ RA,^[25]^ SLE,^[26]^ and type 1 diabetes^[27]^ were obtained from the largest available GWAS studies or FinnGen project, respectively. In addition, we obtained the summary genetic associations data for 4 potential confounding factors including obesity, smoking, alcohol drinking, depression, and biological sex from respective GWAS studies. Detailed information on the GWAS datasets used in the study were summarized in Table S1, Supplemental Digital Content, http://links.lww.com/MD/O248.

All the participants of the GWAS studies were of European ancestry. There is no sample overlap between the GWAS populations of the exposure and outcome variables. As all the data used in the MR analyses were publicly available, no ethical approval is required for this study.

### 2.2. Instrumental variables selection

IVs were used as genetic proxy for the exposure variables in the MR analysis. To identify valid IVs, we first extracted the single nucleotide polymorphisms (SNPs) that are significantly associated with the exposures with *P* < 5e−8. Then the SNPs were clumped by the linkage disequilibrium within a 10,000kb window with the threshold of *R*^2^ < 0.001 according to the European populations of the 1000 genomes project.^[28]^ If a SNP was missing in the outcome data, the proxy SNP with *R*^2^ > 0.8 was used alternatively. If no proxy can be identified, this SNP will be removed from the MR analysis. The exposure and outcome data were harmonized with the same effect allele, while the palindromic SNPs with minor allele frequency larger than 0.42 were excluded from the MR analysis to avoid the ambiguity. The strength of the IVs was calculated by the F statistics to meet the requirement of the relevance assumption of MR.^[29]^ In addition, we searched the GWAS catalog^[30]^ to evaluate if the IVs were associated with any potential confounding factors. A sensitivity analysis would be conducted after excluding SNPs that are significantly associated (*P* < 5e−8) with the confounding factors for the requirement of the independence assumption of MR.

### 2.3. Univariable MR analysis

Bidirectional univariable MR analysis was conducted to explore the causal relationships between ADHD and the 8 autoimmune diseases. The random-effect inverse-variance weighted (IVW) method^[29]^ was used the primary method for causal estimates, while weighted median^[31]^ and MR-Egger^[32]^ were used as alternative MR approaches since they are more flexible on the existence of horizontal pleiotropy. The MR-Egger intercept test^[32]^ and MR-PRESSO global test^[33]^ were conducted to check the potential bias by the horizontal pleiotropy for the requirement of the exclusion-restriction assumption of MR. Moreover, we assessed the heterogeneity and stability of the MR estimates by the Cochran *Q* test and leave-one-out analysis, respectively. The Steiger test was used to assess whether the correct causal direction was estimated (i.e. more variance was explained in exposure than outcome by IVs).^[34]^ Given the multiple testing issue, the causal effect was considered significant if the following 3 criteria were met: the IVW *P*-value is less than the Bonferroni corrected threshold (*P* < .05/8 = 0.00625); the directions of causal estimates by the 3 MR approaches were consistent; both MR-Egger intercept test and MR-PRESSO global test indicated nonsignificant horizontal pleiotropy (*P* > .05).

### 2.4. Multivariable MR analysis

For the significant causal relationship indicated by the univariable MR analysis (i.e., ADHD on psoriasis), we further conducted the multivariable MR (MVMR) analysis to evaluate the direct causal effects of ADHD on psoriasis with adjustment for the potential confounding factors. Obesity, smoking, alcohol drinking, and depression were respectively adjusted in the MVMR model since these risk factors for psoriasis^[35–38]^ were strongly associated with ADHD,^[39]^ thus might be the potential confounders of the causal relationship. Moreover, biological sex was adjusted in the MVMR analysis to account for the potential sex-based differences in the causal relationship. The IVW method with the multivariable extension was used for the MVMR analysis.

### 2.5. Mediation analysis

To investigate the potential mediators of the significant causal relationship between ADHD and psoriasis, 2-step mediation analysis was conducted to estimate the mediation effects and the proportion of mediation effects in the total effects. Obesity, smoking, alcohol drinking, and depression were evaluated as the potential mediators as they are found to be linked with both ADHD and psoriasis.^[35–39]^ The causal effects of ADHD on the mediators (β1) and the causal effects of mediators on psoriasis (β2) were estimated using the IVW method respectively. Then the product of the 2 coefficients (β1*β2) was estimated as the mediation effects with delta method to infer the statistical significance.^[40]^

All the statistical analyses were performed using the R packages “TwoSampleMR” (v.0.5.7)^[41]^ and “MR-PRESSO.”^[33]^ The plots were generated using ggplot2 (v3.3.5).

## 3. Results

### 3.1. Causal effects of ADHD on autoimmune diseases by univariable MR analysis

Following the IV selection criteria aforementioned, 7 to 11 independent SNPs robustly associated with ADHD were selected for the MR analysis with the 8 autoimmune diseases. The number of SNPs varies for different autoimmune diseases due to the availability of IVs in the outcome data. All the IVs have F statistics larger than 10 (Table 1), indicating strong instruments.^[29]^ The details of the IVs used in the study were listed in Table S2, Supplemental Digital Content, http://links.lww.com/MD/O248.

**Table 1:** Causal effects of ADHD on autoimmune diseases by univariable MR analysis.

Outcome	nSNP	*F*	Method	OR (95% CI)	*P*	Heterogeneity *P*	MR-Egger intercept *P*	MR-PRESSO global *P*	Correct direction (Steiger *P*)
SLE	9	34.8	IVW	0.99 (0.76–1.3)	9.50e−01	.33	.12	.32	Yes (1.10e−08)
			WM	1.07 (0.75–1.52)	7.00e−01				
			MR-Egger	0.38 (0.13–1.13)	1.30e−01				
CD	9	34.8	IVW	1.02 (0.88–1.18)	8.00e−01	.33	.53	.37	Yes (9.10e−22)
			WM	1.08 (0.9–1.3)	4.00e−01				
			MR-Egger	0.83 (0.43–1.58)	5.80e−01				
UC	9	34.8	IVW	0.98 (0.82–1.17)	7.90e−01	.07	.35	.08	Yes (4.80e−22)
			WM	1.11 (0.92–1.35)	2.70e−01				
			MR-Egger	1.43 (0.67–3.07)	3.90e−01				
T1D	9	34.8	IVW	0.92 (0.72–1.18)	5.10e−01	.14	.49	.11	Yes (2.10e−13)
			WM	1.02 (0.77–1.35)	8.90e−01				
			MR-Egger	0.63 (0.22–1.81)	4.20e−01				
RA	11	34.3	IVW	1.11 (0.97–1.26)	1.30e−01	.11	.5	.14	Yes (3.20e−29)
			WM	1.1 (0.95–1.28)	2.10e−01				
			MR-Egger	1.41 (0.71–2.78)	3.50e−01				
PsO	11	34.3	IVW	1.29 (1.11–1.49)	**6.30e−04**	.96	.88	.95	Yes (2.40e−24)
			WM	1.31 (1.08–1.58)	5.80e−03				
			MR-Egger	1.36 (0.69–2.68)	4.00e−01				
AS	9	34.8	IVW	1.36 (0.99–1.85)	5.50e−02	.45	.88	.47	Yes (4.40e−47)
			WM	1.69 (1.12–2.53)	1.20e−02				
			MR-Egger	1.22 (0.34–4.42)	7.70e−01				
MS	7	35.5	IVW	1.03 (0.86–1.24)	7.60e−01	.23	.92	.29	Yes (5.00e−33)
			WM	1.09 (0.88–1.35)	4.40e−01				
			MR-Egger	0.95 (0.24–3.8)	9.50e−01				

Bold value indicates that IVW *P*-value is less than the Bonferroni corrected threshold (*P* < .05/8 = 0.00625).

AS = ankylosing spondylitis, CD = Crohn’s disease, IVW = Inverse-variance weighted, MS = multiple sclerosis, PsO = psoriasis, RA = rheumatoid arthritis, SLE = systemic lupus erythematosus, T1D = type 1 diabetes, UC = ulcerative colitis, WM = weighted median.

**Figure 2.**
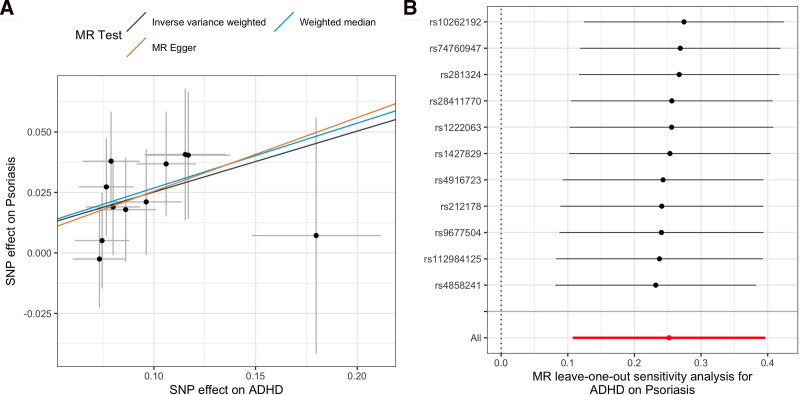
Causal effects of ADHD on psoriasis. (A) Scatter plot of the effects of IVs on ADHD and psoriasis; (B) leave-one-out analysis of the ADHD on psoriasis.

With the Bonferroni corrected threshold, the IVW results indicated that genetically determined higher risk of ADHD was significantly associated with increased risk of psoriasis (IVW OR: 1.29; 95% CI: 1.11–1.49, *P* = 6.3e−04). Estimates by the MR-Egger and weighted median suggested similar results (Fig. 2A). No significant heterogeneity (Cochran *Q* test *P* = .96) and horizontal pleiotropy (MR-Egger intercept test *P* = .88, MR-PRESSO global test *P* = .95) were found, suggesting the robustness of the results. The leave-one-out analysis didn’t identify any SNPs that dominate the causal effects (Fig. 2B), and the Steiger test indicated the direction of the causal relationship was correct (*P* = 2.4e−24). We didn’t find any significant causal effects of ADHD on the other autoimmune diseases (Table 1).

As the observational studies suggested that the relationship between ADHD and autoimmune diseases might be bidirectional, we also conducted the reverse MR analysis with autoimmune diseases as exposures and ADHD as outcomes. The reverse MR analysis didn’t identify any significant causal relationships (Table S3, Supplemental Digital Content, http://links.lww.com/MD/O248).

To assess if the IVs were associated with the confounding factors that might violate the independence assumption of MR, we conducted a phenome-wide associations search using the GWAS catalog database. Two SNPs (rs4916723 and rs4858241) were found to be associated with the potential confounding factors alcohol drinking^[42]^ and smoking,^[43]^ respectively. As shown in Table S4, Supplemental Digital Content, http://links.lww.com/MD/O248, we found similar causal effects of ADHD on psoriasis after removing these 2 SNPs (IVW OR: 1.25; 95% CI: 1.06–1.46, *P* = 6.2e−03), which further ensured the robustness of the results.

### 3.2. Direct causal effects of ADHD on psoriasis by multivariable MR analysis

To evaluate whether the causal effects of ADHD on psoriasis were independent of the potential confounding factors, we conducted the multivariable MR analysis to evaluate the direct causal effects with the adjustment of obesity, smoking, alcohol drinking, depression, and biological sex. We found the significant causal relationship between ADHD and psoriasis remained significant with adjusting for obesity, alcohol drinking, depression, and biological sex (Table 2). However, the causal relationship became nonsignificant when accounting for the effects of smoking initiation, suggesting smoking might be a mediator of the causal relationship.

**Table 2:** Multivariable MR analysis of the causal effects of ADHD on Psoriasis with adjustment of potential confounding factors.

Exposure	Outcome	Adjustment	OR (95% CI)	*P*
ADHD	Psoriasis	Obesity	1.31 (1.13–1.51)	2.10E-04
ADHD	Psoriasis	Smoking initiation	1.08 (0.9–1.3)	4.00E-01
ADHD	Psoriasis	Alcoholic drinks per week	1.27 (1.01–1.6)	4.00E-02
ADHD	Psoriasis	Major depression	1.34 (1.03–1.74)	3.10E-02
ADHD	Psoriasis	Biological sex	1.32 (1.20–1.44)	1.31E-09

ADHD = attention-deficit/hyperactivity disorder.

### 3.3. Mediation analysis to identify potential mediators of the causal relationship between ADHD and psoriasis

Two-step MR mediation analysis was further performed to explore the potential mediating effects of obesity, smoking, alcohol drinking, and depression in the causal relationship between ADHD and psoriasis as these risk factors for psoriasis were also strongly associated with ADHD.^[35–39]^ We found that ADHD increased the risk of smoking initiation, while smoking initiation increased the risk of psoriasis (Table 3). The proportion of the mediating effects of smoking initiation in the total effects was estimated to be 11.16% (95% CI: 1.54% to 20.77%, *P* = .023). We didn’t find significant mediation effects of obesity, alcohol drinking, and depression.

**Table 3:** Mediation MR analysis of the potential mediators of the causal effects of ADHD on Psoriasis.

Mediator	Total effect (ADHD on psoriasis)	Effect 1 (ADHD on mediator)	Effect 2 (mediator on psoriasis)	Mediation effect		Mediated percentage
	Beta (95% CI)	Beta (95% CI)	Beta (95% CI)	Beta (95% CI)	*P*	% (95% CI)
Obesity	0.252 (0.107–0.396)	0.094 (–0.044 to 0.233)	0.062 (–0.082 to 0.207)	0.006 (–0.013 to 0.025)	5.47e−01	2.34 (–5.26 to 9.93)
Smoking initiation	0.252 (0.107–0.396)	0.091 (0.049–0.133)	0.309 (0.09–0.529)	0.028 (0.004–0.052)	2.30e−02	11.16 (1.54–20.77)
Alcoholic drinks per week	0.252 (0.107–0.396)	0.005 (–0.028 to 0.038)	0.109 (–0.421 to 0.638)	0.001 (–0.009 to 0.01)	9.20e−01	0.2 (–3.72 to 4.13)
Major depression	0.252 (0.107–0.396)	0.069 (–0.014 to 0.152)	0.116 (–0.226 to 0.459)	0.008 (–0.021 to 0.037)	5.92e−01	3.19 (–8.48 to 14.87)

ADHD = attention-deficit/hyperactivity disorder.

## 4. Discussions

Through the 2-sample MR approach, we investigated the causal links between ADHD and a range of autoimmune disorders. We found that genetically predicted higher ADHD risk is significantly associated with higher risk of psoriasis, but not with other autoimmune disorders. On the other side, we didn’t find any significant causal effects of autoimmune diseases on the risk of ADHD in the reverse MR analysis. Further multivariable MR and mediation MR analyses suggested that the significant causal relationship between ADHD and psoriasis is independent of the other risk factors such as obesity, alcohol drinking, and depression, but might be partially mediated by the smoking initiation. To the best of our knowledge, this is the first MR study aiming to dissect the causal relationships between ADHD and autoimmune diseases, and to explore the potential underlying mediating pathways.

Our finding of the significant causal relationship between ADHD and psoriasis is consistent with multiple recent observational studies. For example, in a large cross-sectional study investigating the associations between ADHD and multiple autoimmune diseases, the strongest association was found between ADHD and psoriasis regardless of sex.^[11]^ Our MR analysis confirmed the association between ADHD and psoriasis with minimized bias from confounding effects and reverse causation.

Psoriasis is an immune-mediated skin disorder affecting more than 125 million people worldwide.^[44]^ There are several potential pathways that may explain the association between ADHD and psoriasis, although the detailed mechanisms linking these 2 diseases have not been extensively investigated. The shared defective immune regulation might link the 2 disorders. For example, complement system was found to mediate the process of synaptic pruning,^[45,46]^ and the overactive pruning is considered as a key factor leading to the development of ADHD.^[47,48]^ On the other hand, complement activation could initiate the onset of psoriasis through its role in linking the innate and adaptive immune responses.^[49]^ Therefore, it is possible that the relation of the 2 disorders was attributed to the dysregulation of the complement system given that ADHD is increasingly being recognized as an immune-associated disease.^[50]^ The lifestyle and behavior changes associated with ADHD might also contribute to the development of psoriasis. The mediation analysis identified smoking initiation as a significant mediator of the causal relationship between ADHD and psoriasis. It has been well documented that individuals with ADHD are more likely to initiate and continue smoking than people without ADHD,^[51–53]^ while smoking is a well-known risk factor for psoriasis.^[36,54]^ It is likely that the lifestyle changes associated with ADHD such as smoking could trigger the onset of psoriasis.

Our findings of the positive causal relationship between ADHD and psoriasis have important clinical implications. For the individuals especially children diagnosed with ADHD, early screening and management of the psoriasis and related symptoms might be helpful to reduce the risk of onset of psoriasis in their later life. Also, as smoking was identified as a potential mediator, lifestyle changes such as cigarette cessation might be helpful to break the link between ADHD and psoriasis. Further clinical studies were warranted to validate these hypotheses before applying to the clinic.

In our MR analysis, we didn’t observe significant causal relationships between ADHD and other autoimmune diseases. A meta-analysis of observational studies indicated correlation between ADHD and T1D.^[55]^ However, due to the inherent limitations of the observational studies, the correlation might be confounded by the unmeasured environmental and social factors such as diet and medication use. An observational study indicated a positive correlation between ADHD and AS,^[10]^ but another study found the relationship was negative.^[11]^ The inconsistency of the observational studies further highlights the strengths of MR in understand the causal relationships of the correlated conditions.

Our study has several limitations that worth discussing. First, our analysis and findings were restricted to the European populations due to the availability of the data. Further studies were warranted to extend the analysis in the populations with different genetic backgrounds. Second, previous epidemiological studies have revealed the sex differences in the prevalence of the ADHD and autoimmune diseases.^[56,57]^ Our analysis is limited in exploring the sex specific causal effects as the sex stratified GWAS data are rarely available. However, the MVMR analysis with adjustment of biological sex suggested that the causal effects of ADHD on psoriasis are independent of sex. Third, although rigorous sensitivity analyses have been performed to ensure the robustness of the findings, the results are still likely to be biased by horizontal pleiotropy as the inherent limitation of the MR approach.^[58]^

## 5. Conclusions

There is a significant causal relationship between ADHD and psoriasis, but not with other autoimmune disorders. The causal effects might be mediate by smoking. Our findings suggested that early prevention and lifestyle changes (such as smoking cessation) might be helpful to reduce the risk of developing psoriasis for ADHD patients. Further investigations were warranted to explore the underlying mechanisms and the potential clinical applications.

## Author contributions

**Conceptualization:** Yidong Zhou, Bowen Jin, Kai Qiao.

**Investigation:** Yidong Zhou, Bowen Jin, Kai Qiao.

**Methodology:** Yidong Zhou.

**Supervision:** Yidong Zhou.

## Supplementary Material


