# Supplementary material for: Investigating the causal relationships between attention-deficit/hyperactivity disorder and autoimmune diseases: Evidence from Mendelian randomization study
Source: Medicine (Baltimore). 2025 Jan 3;104(1):e41157. doi: 10.1097/MD.0000000000041157 (PMC11709194; doi:10.1097/MD.0000000000041157)
Supplement: Supplementary file 1 [file medi-104-e41157-s001.docx]

**Table S1. GWAS datasets used in the MR analysis**

| **Category** | **Trait** | **ncase** | **ncontrol** | **sample_size** | **Source** | **pmid** | **population** |
| --- | --- | --- | --- | --- | --- | --- | --- |
| Exposures | ADHD | 20183 | 35191 | 55374 | PGC | 30478444 | European |
| Outcomes | Ankylosing spondylitis | 1462 | 164682 | 166144 | FinnGen | 36653562 | European |
|  | Crohn's disease | 12194 | 28072 | 40266 | de Lange KM et al. | 28067908 | European |
|  | Ulcerative colitis | 12366 | 33609 | 45975 | de Lange KM et al. | 28067908 | European |
|  | Multiple sclerosis | 47429 | 68374 | 115803 | International Multiple Sclerosis Genetics Consortium | 31604244 | European |
|  | Psoriasis | 15967 | 28194 | 44161 | Stuart PE et al. | 34927100 | European |
|  | Rheumatoid arthritis | 14361 | 43923 | 58284 | Ha E et al. | 33310728 | European |
|  | Systemic lupus erythematosus | 5201 | 9066 | 14267 | Bentham J et al. | 26502338 | European |
|  | Type 1 diabetes | 9266 | 15574 | 24840 | Forgetta V et al. | 32005708 | European |
| Confounders | Obesity | 8908 | 209827 | 218735 | FinnGen | 36653562 | European |
|  | smoking initiation | 311629 | 321173 | 607291 | GSCAN | 30643251 | European |
|  | Alcoholic drinks per week | NA | NA | 335394 | GSCAN | 30643251 | European |
|  | Major depression | 170756 | 329443 | 500199 | PGC | 30718901 | European |
|  | Biological sex | 245351 | 206951 | 452302 | Pirastu N et al. | 33888908 | European |

PGC: Psychiatric Genomics Consortium; GSCAN: GWAS and Sequencing Consortium of Alcohol and Nicotine use

**Table S2. SNPs used as instrumental variables for the MR analysis of causal effects of ADHD on autoimmune diseases**

| **Exposure** | **Outcome** | **SNP** | **chr** | **pos** | **effect_allele** | **other_allele** | **eaf** | **beta.exposure** | **se.exposure** | **pval.exposure** |
| --- | --- | --- | --- | --- | --- | --- | --- | --- | --- | --- |
| ADHD | Systemic lupus erythematosus | rs10262192 | 7 | 114091753 | A | G | 0.4733 | 0.073204 | 0.0132 | 2.89E-08 |
| ADHD | Systemic lupus erythematosus | rs112984125 | 1 | 44173423 | A | G | 0.2956 | -0.106005 | 0.0146 | 3.58E-13 |
| ADHD | Systemic lupus erythematosus | rs1427829 | 12 | 89760744 | G | A | 0.55 | -0.0799012 | 0.0133 | 1.82E-09 |
| ADHD | Systemic lupus erythematosus | rs212178 | 16 | 72578131 | A | G | 0.8852 | -0.1154 | 0.02 | 7.68E-09 |
| ADHD | Systemic lupus erythematosus | rs281324 | 15 | 47754018 | C | T | 0.462 | 0.0744973 | 0.0134 | 2.68E-08 |
| ADHD | Systemic lupus erythematosus | rs4858241 | 3 | 20669071 | G | T | 0.38 | -0.0789036 | 0.014 | 1.74E-08 |
| ADHD | Systemic lupus erythematosus | rs4916723 | 5 | 87854395 | C | A | 0.4166 | 0.0766003 | 0.0135 | 1.58E-08 |
| ADHD | Systemic lupus erythematosus | rs74760947 | 8 | 34352610 | G | A | 0.0512 | 0.179797 | 0.0317 | 1.35E-08 |
| ADHD | Systemic lupus erythematosus | rs9677504 | 2 | 215181889 | A | G | 0.097 | 0.116903 | 0.0206 | 1.39E-08 |
| ADHD | Crohn's disease | rs10262192 | 7 | 114091753 | A | G | 0.4733 | 0.073204 | 0.0132 | 2.89E-08 |
| ADHD | Crohn's disease | rs112984125 | 1 | 44173423 | A | G | 0.2956 | -0.106005 | 0.0146 | 3.58E-13 |
| ADHD | Crohn's disease | rs1427829 | 12 | 89760744 | G | A | 0.55 | -0.0799012 | 0.0133 | 1.82E-09 |
| ADHD | Crohn's disease | rs212178 | 16 | 72578131 | A | G | 0.8852 | -0.1154 | 0.02 | 7.68E-09 |
| ADHD | Crohn's disease | rs281324 | 15 | 47754018 | C | T | 0.462 | 0.0744973 | 0.0134 | 2.68E-08 |
| ADHD | Crohn's disease | rs4858241 | 3 | 20669071 | G | T | 0.38 | -0.0789036 | 0.014 | 1.74E-08 |
| ADHD | Crohn's disease | rs4916723 | 5 | 87854395 | C | A | 0.4166 | 0.0766003 | 0.0135 | 1.58E-08 |
| ADHD | Crohn's disease | rs74760947 | 8 | 34352610 | G | A | 0.0512 | 0.179797 | 0.0317 | 1.35E-08 |
| ADHD | Crohn's disease | rs9677504 | 2 | 215181889 | A | G | 0.097 | 0.116903 | 0.0206 | 1.39E-08 |
| ADHD | Ulcerative colitis | rs10262192 | 7 | 114091753 | A | G | 0.4733 | 0.073204 | 0.0132 | 2.89E-08 |
| ADHD | Ulcerative colitis | rs112984125 | 1 | 44173423 | A | G | 0.2956 | -0.106005 | 0.0146 | 3.58E-13 |
| ADHD | Ulcerative colitis | rs1427829 | 12 | 89760744 | G | A | 0.55 | -0.0799012 | 0.0133 | 1.82E-09 |
| ADHD | Ulcerative colitis | rs212178 | 16 | 72578131 | A | G | 0.8852 | -0.1154 | 0.02 | 7.68E-09 |
| ADHD | Ulcerative colitis | rs281324 | 15 | 47754018 | C | T | 0.462 | 0.0744973 | 0.0134 | 2.68E-08 |
| ADHD | Ulcerative colitis | rs4858241 | 3 | 20669071 | G | T | 0.38 | -0.0789036 | 0.014 | 1.74E-08 |
| ADHD | Ulcerative colitis | rs4916723 | 5 | 87854395 | C | A | 0.4166 | 0.0766003 | 0.0135 | 1.58E-08 |
| ADHD | Ulcerative colitis | rs74760947 | 8 | 34352610 | G | A | 0.0512 | 0.179797 | 0.0317 | 1.35E-08 |
| ADHD | Ulcerative colitis | rs9677504 | 2 | 215181889 | A | G | 0.097 | 0.116903 | 0.0206 | 1.39E-08 |
| ADHD | Type 1 diabetes | rs10262192 | 7 | 114091753 | A | G | 0.4733 | 0.073204 | 0.0132 | 2.89E-08 |
| ADHD | Type 1 diabetes | rs112984125 | 1 | 44173423 | A | G | 0.2956 | -0.106005 | 0.0146 | 3.58E-13 |
| ADHD | Type 1 diabetes | rs1427829 | 12 | 89760744 | G | A | 0.55 | -0.0799012 | 0.0133 | 1.82E-09 |
| ADHD | Type 1 diabetes | rs212178 | 16 | 72578131 | A | G | 0.8852 | -0.1154 | 0.02 | 7.68E-09 |
| ADHD | Type 1 diabetes | rs281324 | 15 | 47754018 | C | T | 0.462 | 0.0744973 | 0.0134 | 2.68E-08 |
| ADHD | Type 1 diabetes | rs4858241 | 3 | 20669071 | G | T | 0.38 | -0.0789036 | 0.014 | 1.74E-08 |
| ADHD | Type 1 diabetes | rs4916723 | 5 | 87854395 | C | A | 0.4166 | 0.0766003 | 0.0135 | 1.58E-08 |
| ADHD | Type 1 diabetes | rs74760947 | 8 | 34352610 | G | A | 0.0512 | 0.179797 | 0.0317 | 1.35E-08 |
| ADHD | Type 1 diabetes | rs9677504 | 2 | 215181889 | A | G | 0.097 | 0.116903 | 0.0206 | 1.39E-08 |
| ADHD | Rheumatoid arthritis | rs10262192 | 7 | 114091753 | A | G | 0.4733 | 0.073204 | 0.0132 | 2.89E-08 |
| ADHD | Rheumatoid arthritis | rs112984125 | 1 | 44173423 | A | G | 0.2956 | -0.106005 | 0.0146 | 3.58E-13 |
| ADHD | Rheumatoid arthritis | rs1222063 | 1 | 96602440 | A | G | 0.3653 | 0.0962007 | 0.0174 | 3.07E-08 |
| ADHD | Rheumatoid arthritis | rs1427829 | 12 | 89760744 | G | A | 0.55 | -0.0799012 | 0.0133 | 1.82E-09 |
| ADHD | Rheumatoid arthritis | rs212178 | 16 | 72578131 | A | G | 0.8852 | -0.1154 | 0.02 | 7.68E-09 |
| ADHD | Rheumatoid arthritis | rs281324 | 15 | 47754018 | C | T | 0.462 | 0.0744973 | 0.0134 | 2.68E-08 |
| ADHD | Rheumatoid arthritis | rs28411770 | 4 | 31151456 | C | T | 0.3217 | -0.0861043 | 0.0151 | 1.15E-08 |
| ADHD | Rheumatoid arthritis | rs4858241 | 3 | 20669071 | G | T | 0.38 | -0.0789036 | 0.014 | 1.74E-08 |
| ADHD | Rheumatoid arthritis | rs4916723 | 5 | 87854395 | C | A | 0.4166 | 0.0766003 | 0.0135 | 1.58E-08 |
| ADHD | Rheumatoid arthritis | rs74760947 | 8 | 34352610 | G | A | 0.0512 | 0.179797 | 0.0317 | 1.35E-08 |
| ADHD | Rheumatoid arthritis | rs9677504 | 2 | 215181889 | A | G | 0.097 | 0.116903 | 0.0206 | 1.39E-08 |
| ADHD | Psoriasis | rs10262192 | 7 | 114091753 | A | G | 0.4733 | 0.073204 | 0.0132 | 2.89E-08 |
| ADHD | Psoriasis | rs112984125 | 1 | 44173423 | A | G | 0.2956 | -0.106005 | 0.0146 | 3.58E-13 |
| ADHD | Psoriasis | rs1222063 | 1 | 96602440 | A | G | 0.3653 | 0.0962007 | 0.0174 | 3.07E-08 |
| ADHD | Psoriasis | rs1427829 | 12 | 89760744 | G | A | 0.55 | -0.0799012 | 0.0133 | 1.82E-09 |
| ADHD | Psoriasis | rs212178 | 16 | 72578131 | A | G | 0.8852 | -0.1154 | 0.02 | 7.68E-09 |
| ADHD | Psoriasis | rs281324 | 15 | 47754018 | C | T | 0.462 | 0.0744973 | 0.0134 | 2.68E-08 |
| ADHD | Psoriasis | rs28411770 | 4 | 31151456 | C | T | 0.3217 | -0.0861043 | 0.0151 | 1.15E-08 |
| ADHD | Psoriasis | rs4858241 | 3 | 20669071 | G | T | 0.38 | -0.0789036 | 0.014 | 1.74E-08 |
| ADHD | Psoriasis | rs4916723 | 5 | 87854395 | C | A | 0.4166 | 0.0766003 | 0.0135 | 1.58E-08 |
| ADHD | Psoriasis | rs74760947 | 8 | 34352610 | G | A | 0.0512 | 0.179797 | 0.0317 | 1.35E-08 |
| ADHD | Psoriasis | rs9677504 | 2 | 215181889 | A | G | 0.097 | 0.116903 | 0.0206 | 1.39E-08 |
| ADHD | Ankylosing spondylitis | rs10262192 | 7 | 114091753 | A | G | 0.4733 | 0.073204 | 0.0132 | 2.89E-08 |
| ADHD | Ankylosing spondylitis | rs112984125 | 1 | 44173423 | A | G | 0.2956 | -0.106005 | 0.0146 | 3.58E-13 |
| ADHD | Ankylosing spondylitis | rs1427829 | 12 | 89760744 | G | A | 0.55 | -0.0799012 | 0.0133 | 1.82E-09 |
| ADHD | Ankylosing spondylitis | rs212178 | 16 | 72578131 | A | G | 0.8852 | -0.1154 | 0.02 | 7.68E-09 |
| ADHD | Ankylosing spondylitis | rs281324 | 15 | 47754018 | C | T | 0.462 | 0.0744973 | 0.0134 | 2.68E-08 |
| ADHD | Ankylosing spondylitis | rs4858241 | 3 | 20669071 | G | T | 0.38 | -0.0789036 | 0.014 | 1.74E-08 |
| ADHD | Ankylosing spondylitis | rs4916723 | 5 | 87854395 | C | A | 0.4166 | 0.0766003 | 0.0135 | 1.58E-08 |
| ADHD | Ankylosing spondylitis | rs74760947 | 8 | 34352610 | G | A | 0.0512 | 0.179797 | 0.0317 | 1.35E-08 |
| ADHD | Ankylosing spondylitis | rs9677504 | 2 | 215181889 | A | G | 0.097 | 0.116903 | 0.0206 | 1.39E-08 |
| ADHD | multiple sclerosis | rs10262192 | 7 | 114091753 | A | G | 0.4733 | 0.073204 | 0.0132 | 2.89E-08 |
| ADHD | multiple sclerosis | rs112984125 | 1 | 44173423 | A | G | 0.2956 | -0.106005 | 0.0146 | 3.58E-13 |
| ADHD | multiple sclerosis | rs1427829 | 12 | 89760744 | G | A | 0.55 | -0.0799012 | 0.0133 | 1.82E-09 |
| ADHD | multiple sclerosis | rs212178 | 16 | 72578131 | A | G | 0.8852 | -0.1154 | 0.02 | 7.68E-09 |
| ADHD | multiple sclerosis | rs281324 | 15 | 47754018 | C | T | 0.462 | 0.0744973 | 0.0134 | 2.68E-08 |
| ADHD | multiple sclerosis | rs4858241 | 3 | 20669071 | G | T | 0.38 | -0.0789036 | 0.014 | 1.74E-08 |
| ADHD | multiple sclerosis | rs4916723 | 5 | 87854395 | C | A | 0.4166 | 0.0766003 | 0.0135 | 1.58E-08 |

**Table S3. Reverse MR of the causal effects of autoimmune diseases on ADHD**

| **exposure** | **outcome** | **method** | **nsnp** | **OR (95% CI)** | **pval** |
| --- | --- | --- | --- | --- | --- |
| Systemic lupus erythematosus | ADHD | Inverse variance weighted | 39 | 1.01 (0.99 to 1.03) | 0.50 |
| Systemic lupus erythematosus | ADHD | Weighted median | 39 | 0.99 (0.97 to 1.02) | 0.65 |
| Systemic lupus erythematosus | ADHD | MR Egger | 39 | 1.01 (0.97 to 1.06) | 0.55 |
| Crohn's disease | ADHD | Inverse variance weighted | 77 | 1 (0.98 to 1.03) | 0.89 |
| Crohn's disease | ADHD | Weighted median | 77 | 0.99 (0.96 to 1.03) | 0.72 |
| Crohn's disease | ADHD | MR Egger | 77 | 1.02 (0.95 to 1.1) | 0.63 |
| Ulcerative colitis | ADHD | Inverse variance weighted | 53 | 1.01 (0.98 to 1.04) | 0.47 |
| Ulcerative colitis | ADHD | Weighted median | 53 | 1 (0.96 to 1.04) | 0.96 |
| Ulcerative colitis | ADHD | MR Egger | 53 | 0.98 (0.9 to 1.08) | 0.70 |
| Type 1 diabetes | ADHD | Inverse variance weighted | 27 | 1 (0.99 to 1.02) | 0.62 |
| Type 1 diabetes | ADHD | Weighted median | 27 | 1.01 (0.98 to 1.03) | 0.55 |
| Type 1 diabetes | ADHD | MR Egger | 27 | 1.01 (0.99 to 1.04) | 0.39 |
| Rheumatoid arthritis | ADHD | Inverse variance weighted | 74 | 0.99 (0.97 to 1.02) | 0.58 |
| Rheumatoid arthritis | ADHD | Weighted median | 74 | 1 (0.97 to 1.03) | 0.94 |
| Rheumatoid arthritis | ADHD | MR Egger | 74 | 1 (0.97 to 1.04) | 0.96 |
| Psoriasis | ADHD | Inverse variance weighted | 47 | 1 (0.96 to 1.04) | 0.98 |
| Psoriasis | ADHD | Weighted median | 47 | 1.01 (0.97 to 1.06) | 0.64 |
| Psoriasis | ADHD | MR Egger | 47 | 1.01 (0.92 to 1.11) | 0.85 |
| Ankylosing spondylitis | ADHD | Inverse variance weighted | 8 | 1 (0.97 to 1.03) | 0.90 |
| Ankylosing spondylitis | ADHD | Weighted median | 8 | 1 (0.97 to 1.03) | 0.94 |
| Ankylosing spondylitis | ADHD | MR Egger | 8 | 0.98 (0.91 to 1.06) | 0.67 |

**Table S4. Causal effects of ADHD on autoimmune diseases after removing SNPs associated with the confounders**

| **exposure** | **outcome** | **method** | **nsnp** | **OR (95%CI)** | **pval** |
| --- | --- | --- | --- | --- | --- |
| ADHD | Systemic lupus erythematosus | Inverse variance weighted | 7 | 0.86 (0.64 to 1.16) | 0.33 |
| ADHD | Systemic lupus erythematosus | Weighted median | 7 | 0.84 (0.58 to 1.22) | 0.36 |
| ADHD | Systemic lupus erythematosus | MR Egger | 7 | 0.47 (0.15 to 1.46) | 0.25 |
| ADHD | Crohn's disease | Inverse variance weighted | 7 | 0.99 (0.83 to 1.19) | 0.95 |
| ADHD | Crohn's disease | Weighted median | 7 | 1.05 (0.85 to 1.29) | 0.67 |
| ADHD | Crohn's disease | MR Egger | 7 | 0.85 (0.39 to 1.87) | 0.71 |
| ADHD | Ulcerative colitis | Inverse variance weighted | 7 | 0.99 (0.8 to 1.22) | 0.91 |
| ADHD | Ulcerative colitis | Weighted median | 7 | 1.11 (0.89 to 1.37) | 0.36 |
| ADHD | Ulcerative colitis | MR Egger | 7 | 1.44 (0.62 to 3.36) | 0.44 |
| ADHD | Type 1 diabetes | Inverse variance weighted | 7 | 0.88 (0.65 to 1.19) | 0.4 |
| ADHD | Type 1 diabetes | Weighted median | 7 | 1.01 (0.74 to 1.37) | 0.97 |
| ADHD | Type 1 diabetes | MR Egger | 7 | 0.66 (0.19 to 2.35) | 0.55 |
| ADHD | Rheumatoid arthritis | Inverse variance weighted | 9 | 1.17 (1.02 to 1.34) | 0.025 |
| ADHD | Rheumatoid arthritis | Weighted median | 9 | 1.12 (0.95 to 1.32) | 0.18 |
| ADHD | Rheumatoid arthritis | MR Egger | 9 | 1.15 (0.56 to 2.34) | 0.72 |
| ADHD | Psoriasis | Inverse variance weighted | 9 | 1.25 (1.06 to 1.46) | **0.0062** |
| ADHD | Psoriasis | Weighted median | 9 | 1.26 (1.03 to 1.54) | 0.027 |
| ADHD | Psoriasis | MR Egger | 9 | 1.54 (0.75 to 3.16) | 0.28 |
| ADHD | Ankylosing spondylitis | Inverse variance weighted | 7 | 1.4 (0.98 to 2) | 0.063 |
| ADHD | Ankylosing spondylitis | Weighted median | 7 | 1.7 (1.08 to 2.68) | 0.021 |
| ADHD | Ankylosing spondylitis | MR Egger | 7 | 1.08 (0.27 to 4.37) | 0.92 |
| ADHD | multiple sclerosis | Inverse variance weighted | 5 | 1.06 (0.83 to 1.36) | 0.65 |
| ADHD | multiple sclerosis | Weighted median | 5 | 1.13 (0.88 to 1.45) | 0.35 |
| ADHD | multiple sclerosis | MR Egger | 5 | 0.84 (0.15 to 4.62) | 0.86 |
